# Crystal Structure of the FGFR4/LY2874455 Complex Reveals Insights into the Pan-FGFR Selectivity of LY2874455

**DOI:** 10.1371/journal.pone.0162491

**Published:** 2016-09-12

**Authors:** Daichao Wu, Ming Guo, Michael A. Philips, Lingzhi Qu, Longying Jiang, Jun Li, Xiaojuan Chen, Zhuchu Chen, Lin Chen, Yongheng Chen

**Affiliations:** 1 Laboratory of Structural Biology, Key Laboratory of Cancer Proteomics of Chinese Ministry of Health, XiangYa Hospital, Central South University, Changsha, Hunan 410008, China; 2 Molecular and Computational Biology, Department of Biological Sciences, University of Southern California, Los Angeles, CA 90089, United States of America; 3 Collaborative Innovation Center for Cancer Medicine, Guangzhou, 510060, Guangdong, China; NCI at Frederick, UNITED STATES

## Abstract

Aberrant FGFR4 signaling has been documented abundantly in various human cancers. The majority of FGFR inhibitors display significantly reduced potency toward FGFR4 compared to FGFR1-3. However, LY2874455 has similar inhibition potency for FGFR1-4 with IC50 less than 6.4 nM. To date, there is no published crystal structure of LY2874455 in complex with any kinase. To better understand the pan-FGFR selectivity of LY2874455, we have determined the crystal structure of the FGFR4 kinase domain bound to LY2874455 at a resolution of 2.35 Å. LY2874455, a type I inhibitor for FGFR4, binds to the ATP-binding pocket of FGFR4 in a DFG-in active conformation with three hydrogen bonds and a number of van der Waals contacts. After alignment of the kinase domain sequence of 4 FGFRs, and superposition of the ATP binding pocket of 4 FGFRs, our structural analyses reveal that the interactions of LY2874455 to FGFR4 are largely conserved in 4 FGFRs, explaining at least partly, the broad inhibitory activity of LY2874455 toward 4 FGFRs. Consequently, our studies reveal new insights into the pan-FGFR selectivity of LY2874455 and provide a structural basis for developing novel FGFR inhibitors that target FGFR1-4 broadly.

## Introduction

The human fibroblast growth factor receptor (FGFR) family of proteins (FGFR1-4) contain an extracellular domain, a transmembrane domain, and a cytoplasmic kinase domain [1]. The FGFR family has critical roles in development and tissue repair through the initiation of multiple signaling cascades controlling proliferation, migration and survival [2, 3]. The FGFR signal pathway is activated through FGF binding to the extracellular domain of FGFR, resulting in dimerization of FGFR molecules. Subsequently, the cytoplasmic kinase of FGFR phosphorylates FGFR substrate 2 (FRS2) and initiates downstream signaling with the activation of the phosphoinositide-3-kinase (PI3K)/AKT and mitogen activated protein kinase (MAPK) pathways [4]. The PI3K/AKT pathway regulates motility and survival, and the MAPK pathway regulates proliferation and migration [5]. FGFR signaling also couples with phospholipase C-gamma (PLC-γ) in a FRS2-independent manner and stimulates protein kinase C (PKC), which partially reinforces the MAPK pathway activation by phosphorylating RAF [3, 4].

FGFR4 signal pathway is strictly controlled under different physiological states. Aberrant FGFR4 signaling pathways, resulting from gene mutation [6], amplification [7] or overexpression [8], play an important role in the proliferation, survival and metastasis of a variety of cancer cells [9, 10]. Some mutations in the FGFR4 kinase domain (N535K and V550E) lead to sustained activation of the FGFR4 signaling pathway in rhabdomyosarcoma [6], malignant lung adenoma and glioma [4, 11, 12]. Additionally, the Y367C mutation, which is outside of the FGFR4 kinase domain, causes FGFR4 to form a homo-dimer spontaneously, resulting in the constitutive activation of the FGFR4 signaling pathway in MDA-MB453 breast cancer cells [13]. Moreover, FGFR4 amplification or overexpression, which are associated with a poor clinical prognosis, are observed in hepatocellular carcinoma (HCC), breast cancer, colon cancer, pancreatic cancer, prostate cancer, and neuroastrocytoma [8, 14–17]. For example, as many as 33% of HCC patients and 32% of breast cancer patients have FGFR4 overexpression [8].

**Fig 1:**
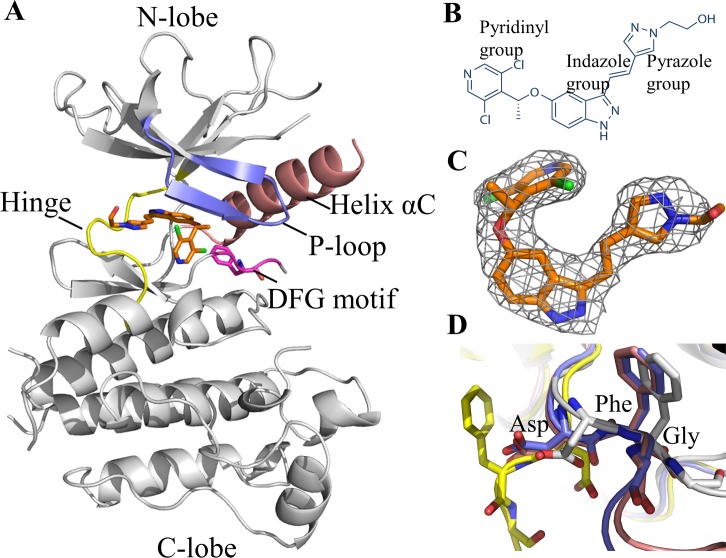
Structure of LY2874455 in complex with FGFR4. **A:** Overall structure of LY2874455/FGFR4 complex. **B:** The diagram of LY2874455. **C:** Fo-Fc omit map of LY2874455 in the FGFR4/LY2874455 complex. The electron density is superimposed with the final model. **D:** The DFG motif conformation of FGFR4. Active ^Apo^FGFR4 DFG-in conformation is shown in blue (PDB: 4QQT); FGFR4/Ponatinib DFG-out conformation is shown in yellow (PDB: 4UXQ); FGFR4/BLU9931 DFG-in conformation is shown in pink (PDB: 4XCU); FGFR4/LY2874455 DFG-in conformation is shown in grey (this work). LY2874455 is highlighted in brown.

To interrupt the aberrant FGFR4 signaling pathway, competitive inhibition of the kinase activity of FGFR has been shown to be an effective method. A number of small molecule ATP-competitive inhibitors for FGFRs, such as pan-FGFR inhibitors PD173074, LY2874455, Ponatinib, CH5183284, BGJ398 and AZD4547, are currently being pursued in the clinic for FGFR-associated cancers. [18–23]. Due to the high similarity of the kinase domains among FGFR1-3 and the fact that FGFR4 has diverged from the other three members, the majority of reported small-molecule FGFR inhibitors, such as CH5183284, BGJ398 and AZD4547, display a significantly reduced potency toward FGFR4 compared to FGFR1-3 [24]. For example, BGJ398, a selective inhibitor of FGFR tyrosine kinase currently in phase II clinical trials for the treatment of FGFR-dependent tumors, has a half maximal inhibitory concentration (IC50) for FGFR1-3 at a single digit nanomolar level, whereas its IC50 for FGFR4 is more than 40-fold higher [22]. However, LY2874455 (Fig 1B) has similar inhibition potency for 4 FGFRs with IC50 less than 6.4 nM in biochemical assays, and is currently in phase I clinical trials for the treatment of FGFR-dependent tumors. It exhibits a potent, FGFR-dependent anti-proliferative activity [18].

Although numerous crystal structures of FGFRs in complex with inhibitors have been resolved [20, 21, 25–27], no crystal structure of LY2874455 in complex with any kinase has been reported. The mechanisms of pan-FGFR selectivity of LY2874455 remain unknown. In this study, we have determined the crystal structure of the FGFR4 kinase domain bound to LY2874455 at a resolution of 2.35 Å. Our studies reveal new insights into the pan-FGFR selectivity of LY2874455 and provide a structural basis to further design and develop new potent pan-FGFR4 inhibitors.

## Results and Discussion

### LY2874455 binds to the ATP-binding pocket of FGFR4 in a unique conformation

To understand how the anti-cancer drug LY2874455 interacts with the kinase domain of FGFR4, we determined the X-ray structure of the FGFR4 kinase domain in complex with LY2874455 with a resolution of 2.35 Å. The crystal belongs to the space group *P*4_3_2_1_2 with cell dimensions of a = 61.89 Å, b = 61.89 Å, c = 186.06 Å. The statistics of data collection are listed in Table 1. The asymmetric unit of the crystal contains one drug molecule, LY2874455. The chemical structure of LY2874455 is shown in Fig 1B. The electron density of LY2874455 is well-defined in the crystal structure (Fig 1C) except for the ethanol group, which appears to be flexible even in the protein-bound state. The FGFR4 kinase domain adopts the typical bi-lobed architecture with an N-terminal lobe (N-lobe) and a C-terminal (C-lobe). The ATP-binding site is located at the cleft between the N- and C- lobes, surrounded by the Hinge region, P-loop, Helix αC, and activation loop (Fig 1A). Part of the activation loop (amino acids 634–650, not included in the coordinates of the final model) exhibits no electron density, indicating that this region is flexible. On the other hand, the DFG motif and the P-loop both have well-determined electron density.

**Table 1:** Data collection and refinement statistics.

	FGFR4/LY2874455
**Wavelength (**Å**)**	0.9789
**Resolution range (**Å**)**	31.89–2.35 (2.44–2.35)
**Space group**	*P* 4_3_2_1_2
**Unit cell**	61.89, 61.89, 186.06, 90, 90, 90
**Total reflections**	440053 (44001)
**Unique reflections**	15694 (1524)
**Multiplicity**	28.0 (28.9)
**Completeness (%)**	99.1 (98.8)
**Mean I/sigma(I)**	50.74 (8.47)
**Wilson B-factor**	49.57
**R-merge**	0.079 (0.756)
**R-meas**	0.081 (0.734)
**CC1/2**	1.000 (0.971)
CC*	1.000 (0.993)
***R*-work**	0.233 (0.253)
***R*-free**	0.277 (0.322)
**Number of non-hydrogen atoms**	2171
** macromolecules**	2103
** ligands**	30
** water**	38
**Protein residues**	266
**RMS deviation (bonds)**	0.010
**RMS deviation (angles)**	1.39
**Ramachandran favored (%)**	92.0
**Ramachandran outliers (%)**	3.9
**Molprobity Clashscore**	16.44
**Average *B*-factor**	66.8
** macromolecules**	67.00
** ligands**	65.40
** solvent**	57.60

The DFG motif is located at the N-terminal of the activation loop, and its conformation plays a pivotal role in kinase activity. DFG-in and DFG-out conformations represent two extreme states of a continuum of possibilities [28]. The DFG-in conformation indicates an active and open conformation of the kinase domain, as seen in the structures of ^Apo^FGFR4 (PDB: 4QQT) [21] and FGFR4/BLU9931 complex (PDB: 4XCU) [26]. The DFG-out conformation indicates an inactive and closed conformation of the kinase domain, as seen in the structures of the FGFR4/Ponatinib complex (PDB: 4UXQ) [25]. We compared the DFG motif conformation in our structure of the FGFR4/LY2874455 complex with that of ^Apo^FGFR4 and FGFR4/Ponatinib. Apparently, upon LY2874455 binding, the FGFR4 kinase domain was kept in DFG-in conformation (Fig 1D).

It is well known that the kinase domain adopts different conformations upon binding to different inhibitors. These inhibitors are are typically divided into three groups referred to as type I, type II, and type III [29]. Type I inhibitors do not induce any conformational change. For example, AZD4547 occupies the ATP-binding pocket, and adopts a DFG-in conformation of the catalytically important DFG motif [20]. Type II inhibitors, such as Ponatinib, occupy not only the ATP-binding site, but also extend beyond the DFG-motif and occupy an additional region, resulting in the DFG motif rotating by 180° in the DFG-out conformation [25]. Type III inhibitors bind to the regulatory domain of the kinase beyond the ATP-binding pocket and modulate the kinase activity allosterically. However, there is no allosteric Type III inhibitor that targets the kinase domain of FGFRs. In the FGFR4/LY2874455 complex, LY2874455 only occupies the ATP-binding site, and does not induce any conformational changes, which indicates that LY2874455 is a Type I inhibitor for FGFR4.

**Fig 2:**
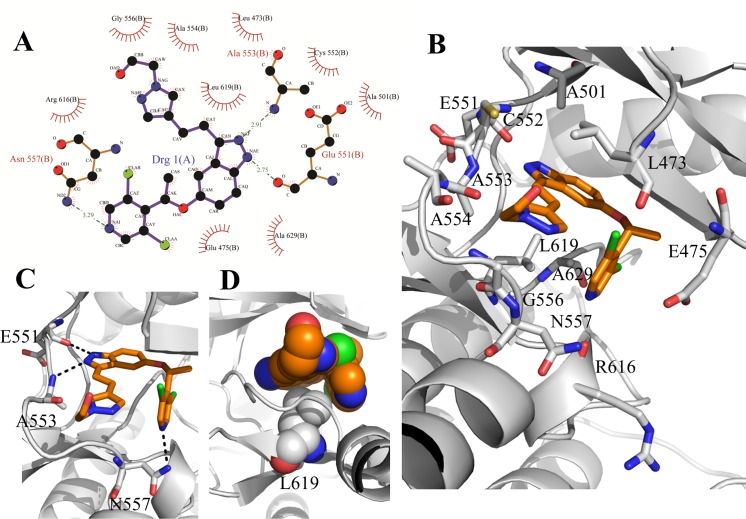
The interactions of LY2874455 with FGFR4. **A:** Schematic diagram of protein-ligand interactions in FGFR4/LY2874455 complex. Hydrogen bonds are indicated by dashed lines between the atoms involved, while hydrophobic contacts are represented by an arc with spokes. The diagram was generated by PDBsum. **B:** The binding conformation of FGFR4 in complex with LY2874455. The side chain conformations of twelve residues interacting with LY2874455. C: LY2874455 binds in the ATP-binding cavity of FGFR4 with three hydrogen bonds. D: The hydrophobic contacts between LY2874455 and Leu619 of FGFR4.

LY2874455 binds to the ATP pocket of FGFR4 through extensive interactions (Fig 2A). It is orientated such that the indazolyl ring points toward the N-terminal part of the Hinge region, and the dichloropyridinyl ring points toward the C-terminal part of the Hinge region, while the ethanol group points outward from the FGFR4 ATP-binding pocket. The indazolyl ring forms two hydrogen bonds with residues in the N-terminal Hinge region, one with the main-chain carbonyl group of glutamic acid (Glu551) and the other with the main-chain amide of alanine (Ala553). The dichloropyridinyl ring forms a hydrogen bond with the side chain of asparagine (Asn557) in the C-terminal Hinge region (Fig 2C). In addition to the hydrogen bonds, LY2874455 also forms a number of van der Waals contacts with 12 residues within ATP-binding pocket of FGFR4 kinase (Fig 2A and 2B). Specifically, Glu475 in the P-loop forms six hydrophobic contacts with the ethoxyl of the inhibitor, while Ala553 and Gly556 in the Hinge region form five and seven hydrophobic contacts with LY2874455, respectively. Moreover, LY2874455 in the ATP-binding pocket adopts a chair-like conformation that folds up on the hydrophobic residue Leu619 in the C-lobe making five hydrophobic contacts, and exhibiting remarkable shape and chemical complementarities between the small molecular drug and the kinase protein (Fig 2D).

### Structural basis of the pan-selectivity of LY2874455 to FGFRs

**Fig 3:**
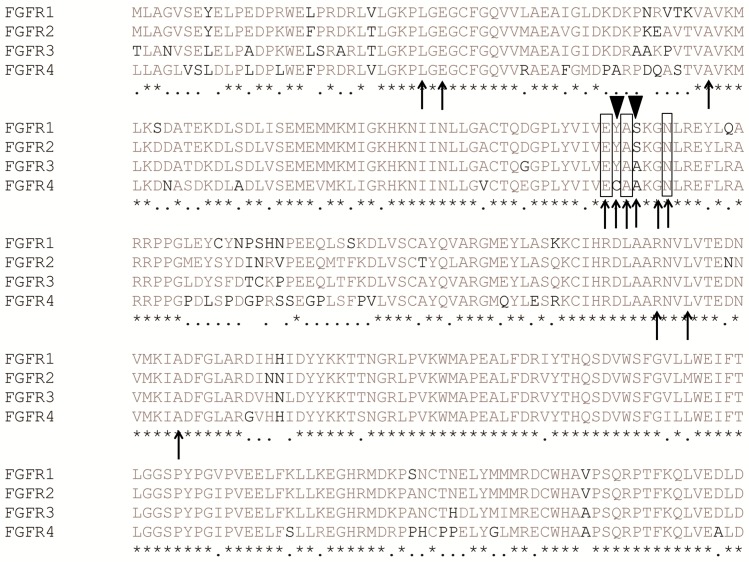
Alignment of 4 FGFR kinase domain sequences. Residues forming hydrogen bonds with LY2874455 are highlighted in black boxes; Residues forming hydrophobic contacts with LY2874455 are highlighted with black arrows. Nonconserved residues in the ATP-binding pocket of FGFRs are highlighted with black triangles.

To understand the mechanism of the pan-selectivity of LY2874455 to FGFRs, we analyzed the hydrogen bonds, hydrophobic contacts, and binding conformation of LY2874455 between FGFR isoforms. Initially, we aligned the kinase domain sequences of the FGFR family (FGFR1-4), which revealed a high sequence similarity between the four closely related proteins. Ten out of twelve FGFR4 hydrophobic contacts with LY2874455 are consistent with FGFR1-3, except for C552 and A554 residues (Fig 3). In particular, key LY2874455-binding residues of FGFR4, such as Glu551, Ala553, Asn557 and Leu619, are all conserved in FGFR1-3 (Fig 3), suggesting that the binding mode of LY2874455 to FGFR1-4 may be conserved in FGFR1-3.

**Fig 4:**
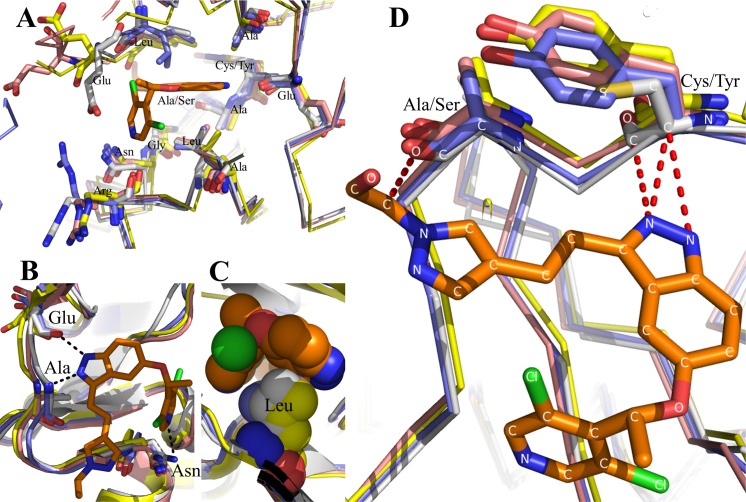
Molecular interactions of LY2874455 with 4 FGFRs. Structural superimposition of FGFR4/LY2874455 complex with other FGFR/drug complexes. **A:** The similarity of twelve-amino acid side chains of 4 FGFRs interacting with LY2874455. **B**: The hydrogen bond-binding sites of LY2874455 to FGFR4 are identical to the corresponding sites of FGFR1-3. Hydrogen bonds are shown in black dashed lines. **C**: The conformation of hydrophobic residue Leu619 in C-lobe of FGFR4 contacts with LY2874455 are conserved in FGFR1-3. **D:** The similar hydrophobic contacts between LY2874455 and FGFR nonconserved residues. Hydrophobic contacts are shown in red dashed lines. FGFR4 is shown in light grey; ^Apo^FGFR1 is shown in blue (PDB: 4UWY); FGFR2 is shown in pink (PDB: 2PSQ); FGFR3 is shown in yellow (PDB: 4K33); FGFR4 is shown in grey (our data). Inhibitor LY2874455 is highlighted in brown.

Since there is no structural information of LY2874455 bound to FGFR1-3, we superimposed the structure of FGFR1-3 to that of FGFR4 in the LY2874455/ FGFR4 complex. The result shows that ten conserved residues occupy a similar spatial position in 4 FGFRs and engage in similar interactions with LY2874455 (Fig 4A). Specifically, the binding conformations of three hydrogen-bonding residues are conserved in 4 FGFRs (Fig 4B). Most notably, Leu619 in FGFR4 and the corresponding Leu in FGFR1-3, are in a position to make extensive hydrophobic and van der Waals contacts with LY2874455 in perfect shape complementarity (Fig 4C). Although C552 and A554 residues in the ATP-binding pocket of FGFR4 are not conserved in other 3 FGFRs, the contacts between ligand and residues are conserved, as evident by the fact that the main-chain carbon atoms of C552 form three hydrophobic contacts with the indazole nitrogen atoms of LY2874455, and the carbonyl of A554 form hydrophobic contacts with the ethanol of LY2874455 (Fig 4D). Therefore, the binding mode of LY2874455 to FGFR1-3 is likely to be similar to that to FGFR4. The intimate and extensive interactions observed in our crystal structure may explain, at least partly, the high binding affinity and potent inhibition of LY2874455 to 4 FGFRs.

LY2874455 is a relatively small inhibitor with a molecular weight of 444.31 g/mol. We can design further derivatives based on the scaffold of LY2874455 to improve the potent inhibitory activity toward FGFR family kinases, such as introducing additional chemical groups to LY2874455, which interact with FGFRs conserved amino acids within the ATP-binding cavity of FGFRs. Furthermore, adding additional function groups to the pyridinyl group of LY2874455 to change the DFG motif conformation to turn it into a type II inhibitor might improve the affinity with FGFR kinases.

On the other hand, low selectivity inhibitors of FGFR4 can lead to side effects due to off-target action, such as hyperphosphatemia, onycholysis, mucositis, and so on [30]. To improve the FGFR4 selectivity and reduce the side effects of inhibitors, it is necessary to design further derivatives based on the scaffold of LY2874455. Because the ATP-binding pocket of FGFR1-4 is highly conserved, it may be difficult to improve the FGFR4 selectivity substantially using the above design approaches, and, therefore, other structural features may need to be explored. One such structural feature is the central Hinge where FGFR1-3 are tyrosine whereas FGFR4 has a cysteine at the corresponding position. So, designing inhibitors to target the sulfhydryl of cysteine in FGFR4 directly could improve the selectivity for FGFR4. Recently, Hagel and colleagues reported an FGFR4-specific kinase inhibitor BLU9931 for treating several models of HCC. The acrylamide of BLU9931 forms a covalent bond with Cys552 of FGFR4 and exhibits a potent and selective inhibition for FGFR4 (IC50 is 3 nM), while displaying only weak activity against FGFR1-3 (IC50 values range from about 150 to 600 nM) [26].

## Conclusions

In this paper, we have determined the first crystal structure of LY2874455 in complex of FGFR4 kinase domain. LY2874455 binds to the ATP-binding pocket of FGFR4 in a unique, chair-like conformation through a multitude of interactions. By structural comparison with other FGFR/drug complexes and structure-guided sequence comparison of FGFR1-4, our studies reveal that the interactions and binding conformation of LY2874455 to FGFR4 might be similar to FGFR1-3. These structural observations explain the observed broad-spectrum and potent inhibitory activity of LY2874455 toward FGFR family kinases. Summarily, our structural studies reveal new insights into the molecular mechanisms of pan-FGFR specificity of LY2874455 and provide a structural basis to further design and develop new potent pan-FGFR inhibitors.

## Methods

### Protein expression and purification

The expression and purification procedure of the kinase domain of human FGFR4 was performed as previously described [21, 25]. In brief, the kinase domain of FGFR4 (residues 445–753) was cloned into modified pET28a to construct the recombinant vector, which contains a PresCission protease-cleavable N-Terminal 6×His tag. The mutation (C477A) was constructed using the QuikChange site-directed mutagenesis kit. After the recombinant vector was transformed into *E*. *coli* BL21 Rosetta, recombinant FGFR4 (rFGFR4) was induced by addition of 0.2 mM IPTG to a shaker flask at 16°C for 20 hours when the culture grew to an OD600 of approximately 0.8. Cell pellets were gathered at 4°C by 10 000 g centrifugation and re-suspended in 10 volumes of buffer A (50 mM Tris-HCl, pH 8.0, 500 mM NaCl, 20 mM imidazole) supplemented with protein inhibitors (PMSF, Leupeptin hemi-sulfate salt, and Pepstatin A), and then lysed by ultrasound pyrolysis. The lysates were clarified by centrifugation for 30 min at 4°C. The lysate supernatants were incubated with equilibrated Ni-NTA beads (GE Health Company) at 4°C for 1 h. The beads were loaded into a gravity flow column, and washed with buffer A containing 50 mM imidazole. The target proteins were eluted with buffer A containing 250 mM imidazole. The N-Terminal His-tag was cleaved by PresCission protease at 4°C for overnight. Then, rFGFR4 was further purified by anion exchange chromatography (Mono Q). The target protein was eluted with a 0–1 M NaCl gradient. The purified rFGFR4 was concentrated to about 10 mg/ml before snap frozen in liquid nitrogen and stored at -80°C for later use in biophysical studies.

### Crystallization

The rFGFR4 was thawed on ice. Inhibitor LY2874455 was purchased from Selleckchem (Beijing, China). To generate FGFR4/LY2874455 co-crystals, rFGFR4 and LY2874455 were mixed at a molar ratio of 1:2 on ice for 30 min before crystallization screen. The FGFR4/LY2874455 crystals were obtained at 4°C using the hanging drop vapor diffusion method in a buffer composed of 0.65 M NH_4_H_2_PO_4_ [20]. Crystals grew in about 3 days and were cryoprotected in 0.7 M NH_4_H_2_PO_4_ plus 20% glycerol, and then flash frozen in liquid nitrogen for later use in data collection.

### Data collection and structure determination and analysis

Data were collected at the Shanghai Synchrotron Radiation Facility (SSRF), beamline BL17U and reduced using HKL3000. Structure determination was carried out as described previously [31, 32]. Initial phase determination was performed by molecular replacement with Phaser from the CCP4 package, using the previously solved FGFR4/Ponatinib structure (PDB code 4TYJ) as the search model [20]. The structure was refined using Phenix.refine and Coot from the Phenix package. The statistics of the crystallographic analysis are presented in Table 1. Graphical representations of structure were prepared using PyMol. The kinase domain sequences of 4 FGFRs were aligned by Clustal X software [33]. The diagram of protein-ligand interaction was generated using PDBsum (www.ebi.ac.uk/thornton-srv/databases/pdbsum).

#### Accession number

Coordinates and structure factors have been deposited in the Protein Data Bank with accession number 5JKG.
